# Case Report: Subclinical Verminous Pneumonia and High Ambient Temperatures Had Severe Impact on the Anesthesia of Semi-domesticated Eurasian Tundra Reindeer (*Rangifer tarandus tarandus*) With Medetomidine–Ketamine

**DOI:** 10.3389/fvets.2021.606323

**Published:** 2021-02-24

**Authors:** Morten Tryland, Terje D. Josefsen, Javier Sánchez Romano, Nina Marcin, Torill Mørk, Jon M. Arnemo

**Affiliations:** ^1^Arctic Infection Biology, Department of Arctic and Marine Biology, UiT The Arctic University of Norway, Tromsø, Norway; ^2^Section for Research in Food Safety and Animal Health, Norwegian Veterinary Institute, Tromsø, Norway; ^3^Faculty of Biosciences and Aquaculture, Nord University, Bodø, Norway; ^4^Department of Forestry and Wildlife Management, Inland Norway University of Applied Sciences, Evenstad, Koppang, Norway; ^5^Department of Wildlife, Fish and Environmental Studies, Swedish University of Agricultural Sciences, Umeå, Sweden

**Keywords:** Alfa-2 agonist, atipamezole, chemical immobilization, circulation, ketamine, medetomidine, parasite, rangifer (reindeer/caribou)

## Abstract

Semidomesticated Eurasian tundra reindeer (*Rangifer tarandus tarandus, n* = 21) were scheduled twice for chemical immobilization with medetomidine–ketamine as part of a scientific experiment in June 2014. During the first round of immobilizations, seven animals developed severe respiratory depression (RD). Three individuals died, and 4 recovered. The ambient temperature during the 2 days of immobilization (June 3 and 4) was high (mean 13.9–17.6°C) compared to the normal mean temperature for these 2 days (7–8°C) based on statistical records. During the second round of immobilizations, using the same anesthetic protocol for the remaining animals as in the first round but conducted under cooler conditions (mean 6.6°C for the period June 9–18), no signs of RD were observed. Clinical and pathological investigations indicated that the animals suffered from circulatory changes possibly caused by high ambient temperatures and granulomatous interstitial pneumonia due to *Elaphostrongylus rangiferi* larvae. These conditions, together with the cardiovascular effects of medetomidine, were likely causes of RD and the fatal outcome. We conclude that chemical immobilization of reindeer with medetomidine–ketamine should be avoided in May–June due to the potential risk when animals partly in winter coats encounter rising ambient temperatures and usually have parasites developing in their airways.

## Background

A combination of medetomidine and ketamine is commonly used for chemical immobilization of reindeer and caribou (*Rangifer tarandus* ssp.) and has been the recommended anesthetic drug combination for Eurasian tundra reindeer (*Rangifer tarandus tarandus*) for more than 20 years (1, 2). Medetomidine, a potent alpha-2 adrenoceptor agonist, has sedative, analgesic, and muscle relaxing properties, whereas ketamine is a dissociative anesthetic drug (3). In combination, medetomidine–ketamine induces dose-dependent immobilization or anesthesia. A commonly encountered adverse effect of medetomidine–ketamine in reindeer is respiratory depression (RD), with subsequent hypoxemia and hypoxia (2). However, we are not aware of any studies reporting severe adverse effects or mortalities in adult reindeer during immobilization with medetomidine–ketamine.

Here, we report high rates of severe morbidity and mortality due to RD in reindeer immobilized with recommended doses of medetomidine–ketamine and discuss possible causes and risk factors based on the environmental condition, clinical signs, and necropsy findings.

## Case Descriptions

### Animals

One-year-old semidomesticated reindeer (*n* = 21), 5 non-pregnant females and 16 males, were selected for an experimental study and brought from a mountain pasture to a corral [for details, see Tryland et al. (4)]. Body condition generally increased during the habituation period (May 6–June 2, 2014). Mean body mass at the time of immobilization (weighing of chemically immobilized animals) was 48.8 kg (SD 6.5, range 32–59). Based on behavior and observations during the habituation period, all animals were apparently healthy, although transient coughing (days and weeks before immobilization) was noted for 13 animals, but most severe (>1–2 days) for animals R11, R12, and R14, which all experienced RD and succumbed from anesthesia [respiratory depression with fatal outcome (RDF)]. Some coughing was also noted for animals R1, R6, and R19, which developed severe RD during anesthesia, and for six of the 14 remaining animals, in which RD was not observed.

### Chemical Immobilization

Animals were chemically immobilized by dart injection from ~11 to 12 m using a CO_2_-powered rifle (JM Special, Dan-Inject ApS, Børkop, Denmark), 3-ml plastic syringes, and 2 × 30 mm barbed needles with side-ports (Dan-Inject ApS). All animals were darted in the large muscles of the lateral thigh while calmly grazing in a corral. A combination of medetomidine (Zalopine 10 mg/ml, Orion Corporation Animal Health, Espoo, Finland) and ketamine (Ketalar 50 mg/ml, Pfizer AS, Oslo, Norway) was used. Initial dose ranges were 3.5–9.0 mg medetomidine and 17.5–45.0 mg ketamine per animal, with a fixed ratio of 1:5 (mg:mg) for medetomidine:ketamine. Body mass was not known prior to immobilization. Doses in the high range were used in the first 5 animals based on Ryeng et al. (1), as recommended for darting. Two of these animals developed signs of RD during anesthesia, and subsequent doses were adjusted to lower doses, as recommended for hand injection (1). Since RD continued to occur in a few animals, doses were further reduced (Table 1). Immobilized animals were weighed in a tarpaulin using a spring scale. The mean drug doses were 0.116 (SD 0.051; range 0.063–0.203) mg/kg medetomidine and 0.578 (SD 0.256; range 0.313–1.014) mg/kg ketamine. The mean time from darting to the animal laid down on the ground [time body down (TBD)] was 4 min 5 s (SD 1 min 58 s, range 1 min 35 s−9 min 31 s). The mean time from darting to when the head was on the ground was 5 min 21 s (SD 1 min 56 s, range 2 min 46 s−9 min 50 s). Animals were maintained in sternal recumbency. Observations of clinical parameters were recorded at ~5, 15, and 30 min after darting, before the animals were placed in smaller corrals for each experimental group. Rectal temperature was measured with a digital thermometer. Pulse rate and peripheral oxygen hemoglobin saturation were measured by pulse oximetry (SpO_2_; Masimo Rad 5v Pulsoksimeter; Infiniti Medical, Drammen, Norway), with the sensor applied to the tongue. Respiratory rate was recorded by observing thoracic wall movements and using a stopwatch.

**Table 1:** Individual recording of the seven animals that experienced respiratory depression (RDF, respiratory depression fatal; RDR, respiratory depression, recovered) compared to the mean for the remaining 14 animals (RD–, no respiratory depression; mean values, *n* = 14, bottom row): body mass, drug doses, time from immobilization to body down (TBD) and head down (THD), heart rate, arterial oxyhemoglobin (SpO_2_), respiratory rate, and rectal temperature recorded during sedation.

**Animal group**	**Animal ID**	**Sex**	**Body mass kg**	Dose Medet + ket^*^ mg/kg	**TBD Min:s**	**THD Min:s**	Heart rate^**^ Beats/min	SpO_2_^**^ %	Resp. rate^**^ Breaths/min	Rectal temperature^**^°C
RDF	11	M	50	0.08 + 0.40	3:46	5:53	75	71	20	39.4
	12	M	47	0.07 + 0.37	3:01	5:18	83	75	40	39.5
	14	F	43	0.17 + 0.87	3:27	3:27	62	65	–	39.8
	Mean RDF	–	47	0.11 + 0.55	3:25	4:53	73	70	30	39.6
RDR	1	M	50	0.08 + 0.40	2:29 (3:18)	3:48 (5:19)	68 (78)	63 (53)	52 (16)	38.7 (39.1)
	6	M	46	0.20 + 0.98	6:04 (0:45)	6:10 (3:10)	45 (84)	90 (91)	24 (40)	39.4 (39.6)
	8	M	46	0.20 + 0.98	6:05 (0:48)	6:17 (1:08)	77 (62)	79 (81)	8 (24)	39.4 (40.5)
	19	M	46	0.09 + 0.44	3:16 (3:16)	3:48 (4:18)	61 (80)	76 (70)	80 (NR)	39.5 (NR)
	Mean RDR	–	47	0.14 + 0.70	4:29 (2:02)	5:08 (3:29)	63 (76)	77 (74)	41 (27)	39.3 (39.7)
RD-	Mean RD- (*n* = 14)	4 F 10 M	50	0.11 + 0.53	4:07 (4:28)	5:33 (8:15)	58 (48)	82 (86)	28 (43)	39.1 (40.1)

*Numbers in parentheses are recordings during the second round of immobilization; same animals, drugs, and doses, but under significantly lower ambient temperature*.

* *Animals chemically immobilized twice (RDR and RD–) were given the same individual dose both times*.

** *Heart rate, SpO_2_, respiratory rate, and temperature are presented as mean values calculated from 3 measurements during the immobilization (prior to antidote injection or euthanasia). NR, not recorded*.

**Figure 1:**
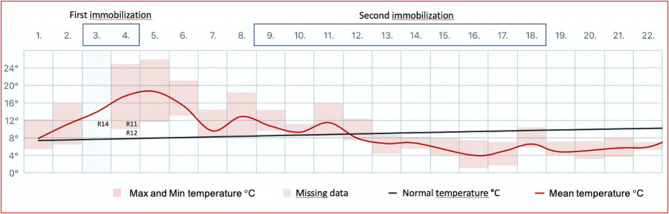
The mean ambient temperature in Tromsø (Holt Meteorological Station; lat. 69.6538, long. 18.9095; Norwegian Meteorological Institute) for June 2014 is shown as a red line. The temperature is measured 100 m above sea level and ~0.5 km from the animal facility. Maximum and minimum temperatures are indicated with pink boxes. Normal temperature is defined as the mean for the reference period 1961–1990. The first round of chemical immobilization (medetomidine–ketamine) of Eurasian tundra reindeer (*Rangifer tarandus tarandus*) was conducted at sunny conditions and under ambient temperatures well-above mean for the period (maximum 17.3°C, mean 13.9°C for June 3; maximum 21.8°C, mean 17.6°C for June 4). The fatal reindeer cases are inserted (R14, R11, R12). In contrast, the second round of immobilization (June 9–18) was conducted under lower temperatures (maximum 11.5°C, mean 6.6°C for the period) and with precipitation as snow.

The first round of immobilization was conducted on June 3–4, 2014, under sunny conditions and with ambient mean temperatures of 13.9–17.6°C, with maximum temperatures of 17.3 and 21.8°C for June 3 and 4, respectively (Figure 1). Atipamezole (Antisedan vet. 5 mg/ml; Orion Corporation Animal Health) at 5 mg/mg medetomidine was administered intramuscularly (i.m.) for reversal 16–36 min (mean 31 min) after darting. Three animals died during the first round of immobilization. For the remaining animals (*n* = 18), the mean times from administration of atipamezole to lifting of the head and standing were 7 min 26 s and 8 min 23 s, respectively.

The second round of immobilization was conducted on June 9–18, 2014, under a mean ambient temperature of 6.6°C, with a maximum temperature of 11.5°C (Figure 1) and with precipitation as snow. Due to the termination of the experiment, the second round of immobilization was followed by euthanasia of the remaining animals (4). The second round of chemical immobilization was carried out with the same drugs and doses as administered for each individual during the first round.

### Clinical Signs and Treatment

During the first round of immobilization, seven of 21 animals showed signs of RD with increased respiratory rate, labored breathing, or apnea, of which 3 animals died. Animal R14 laid down and also laid her head down 3 min 27 s after injection and stopped breathing before we managed to measure respiratory rate. Animals R11 and R12 laid down 3 min 46 s and 3 min 1 s after injection, respectively, but displayed RD and stopped breathing shortly after. The 3 animals (R11, R12, and R14) were treated with a ready-to-use i.m. injection of atipamezole (dose adjusted to the administered dose of medetomidine for each animal) and intravenous injection (i.v.) of 100 mg of the respiratory stimulant doxapram (Dopram 20 mg/ml; Boehringer Ingelheim, Ingelheim am Rhein, Germany). Upon cardiac arrest, these 3 animals were treated with an intracardiac injection of adrenaline (Adrenalin 0.1 mg/ml; Takeda AS, Asker, Norway) as well as cardiac compression. The animals did not respond to any treatment and died (RDF).

Four additional animals (animals R1, R6, R8, and R19) showed increased respiratory rate and labored breathing. Animal R1 received half a dose of atipamezole i.m. and recovered. Animal R6 stopped breathing immediately after the dart injection, received a full dose of atipamezole i.m., and resumed respiration. Animals R8 and R19 received 100 mg doxapram i.v. followed by a full dose of atipamezole i.m. and recovered [respiratory depression, recovered (RDR)].

The remaining 14 animals showed no signs of RD [respiratory depression minus (RD–)] during immobilization. Recordings of body mass, drug dose, time points for TBD and time head down (THD), heart rate, SpO_2_, respiratory rate, and rectal temperature for the two rounds of immobilization are presented in Table 1.

Hyperthermia, defined as body temperature ≥2°C higher than normal (2), was not recorded in any of the animals (Table 1). The recorded rectal temperatures in the RDR and RD– groups when comparing the first (RDR 39.3, RD– 39.1) and the second (RDR 39.7, RD– 40.1) immobilization did not indicate higher rectal temperatures during the first immobilization, conducted during higher ambient temperatures.

### Examination of Carcasses

#### Respiratory Depression With Fatal Outcome Group

**Figure 2:**
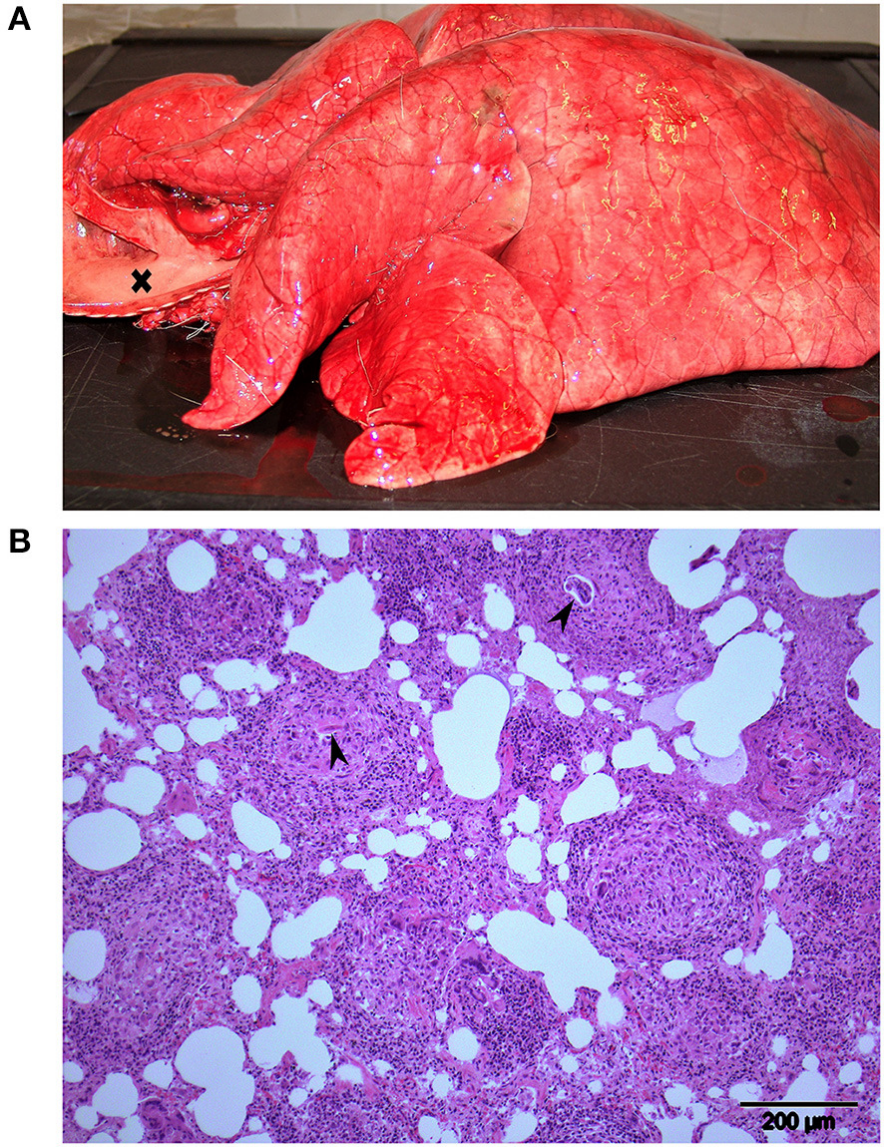
Lungs from animal R12, one of 3 Eurasian tundra reindeer (*Rangifer tarandus tarandus*) that died during chemical immobilization. **(A)** Macroscopic view: Firm rubbery lungs that failed to collapse. Trachea to the left (opened) is filled with foam (cross). **(B)** Histology: Multifocal granulomatous interstitial pneumonia. Developing nematode larvae are visible in 2 granulomas (arrowheads). Approximately 3 quarters of the lung tissue of this reindeer was affected similar to the region shown.

Animals R11, R12, and R14 were subjected to a full necropsy within 24 h after death, including routine histological examination of tissues from myocardium, lung, liver, kidney, and brain. The animals were all in normal body condition for the time of year, with no difference when compared to animals in the RDR and RD– groups. The main gross findings for all 3 RDF animals were in the thorax. Lungs were diffusely firm and rubbery and failed to collapse (Figure 2A). The color of the lungs was light pink, reflecting little blood in lung vessels and capillaries. The interlobular septa were slightly expanded, indicating moderate interstitial edema. Trachea was filled with rich amounts of white or slightly bloodstained viscous foam, oozing out of the mouth and nostrils *post mortem* in animal R12. The heart contained no blood in neither atria nor ventricles. Various amounts of bloodstained subcutaneous edema were present in the thoracic inlet (R12 and R14), lower ventral neck (R12), upper part of the right (R14) or left (R11) shoulder, and in both flanks (R14). There was hyperemia in cutaneous vessels, most pronounced in animal R11. Scattered areas of subcutaneous petechiae were seen in R11 and R12. Histological examination of lung tissue revealed a multifocal, partly coalescing granulomatous interstitial pneumonia in all animals, with frequent occurrence of nematode eggs and developing stages of larvae, morphologically corresponding to *Elaphostrongylus rangiferi* (i.e., “brain worm”) (Figure 2B). The affected lung tissue comprised about 3/4 of the lung tissue in R12, 2/3 in R14, and 1/3 in R11.

#### Respiratory Depression, Recovered, and No Sign of Respiratory Depression Groups

Animals of the RDR group (*n* = 4) and RD– group (*n* = 14), chemically immobilized a second time prior to euthanasia 5–13 days after the initial immobilization, were not subjected to full necropsy. An inspection of body condition and organs was performed, gross findings were subjected to histological examination, and the presence of macroparasites was registered. Diffusely firm and “rubbery lungs” (similar to RDF animals) were observed in R1 (RDR group) and in R2 and R18 (RD– group). In the lungs of R8 (RDR group), firm areas, partly as nodules of 0.5–1.5 cm in diameter, were distributed in cranial and caudal lung lobes on both sides. Histological examination revealed an acute and subacute purulent pneumonia with multifocal necrosis. Bacterial cultivation from lesions showed a rich growth of mixed microbiota, from which *Streptococcus suis II* was isolated.

### Macroparasites in the Respiratory Tract

A low number (1–7) of throat bot larvae (*Cephenemyia trompe*) were detected in two animals from the RDF group and two from the RD– group. Heavy infection with throat bot larvae was found in animal R1 (RDR group), with 107 larvae in the nasopharynx and nasal cavity. Trachea and the main bronchi were opened and inspected in all animals. The reindeer lungworm (*Dictyocaulus eckerti*) was detected in moderate numbers (10–20 worms) in R2 and R20 (RD– group) but not associated with gross pathological changes.

## Discussion and Conclusions

Twenty-one semidomesticated reindeer were anesthetized with medetomidine–ketamine by remote injection. Seven animals displayed increased respiratory rate and labored breathing, and 3 of the animals stopped breathing and died. Necropsy revealed interstitial pneumonia due to *E. rangiferi* larvae. When the remaining animals were anesthetized a second round with the same drugs and individual doses, but under significantly colder weather conditions, no RD was observed.

When two of the animals initially immobilized with the recommended doses displayed RD, measures were taken to avoid complications for the remaining animals. Darting was carried out after 18:00 at lower ambient temperatures and with lower drug doses. Including the first 5 animals that received the full recommended doses for ground-darting of semidomesticated reindeer, the administered doses (*n* = 21) were on average 65% of the recommended dose for medetomidine–ketamine (1). In spite of the reduction, two of the 3 last animals to be immobilized during the first round died. These two individuals received 46% and 43% of the recommended doses, respectively. To investigate if the unexpected severe effects in some animals during the first round of immobilization were associated with the ambient temperatures, we used the same drugs and doses as administered for each individual during the first round in the second round, leaving the temperature difference as the main variable when comparing the two immobilizations.

Mature throat bot larvae that are ejected through the mouth or nostrils of reindeer (late April to June and July) by coughing (5) may cause airway obstruction. Coughing was observed in several animals during the habituation period, but at the time of the second round of immobilization, only 1 animal (R1, RDR group) had a larger number of throat bots. This animal had the lowest SpO_2_ values recorded during both rounds of chemical immobilization (63 and 53%, respectively). Throat bot larvae may thus have influenced blood oxygenation but can hardly explain the RD for other animals hosting only a few larvae.

More importantly, the necropsy findings in the RDF animals suggested circulatory collapse and pulmonary edema. The lungs contained little blood. There was no blood in the heart in any of the 3 RDF animals, which may be due to the conducted cardiac compressions. The spleens in animals R12 and R14 (RDF group) were moderately congested, but otherwise the abdominal organs contained sparse amounts of blood. Thus, the bulk of blood was distributed peripherally, in skin and subcutaneous tissues. This may be a cardiovascular response to high ambient temperatures in order to increase heat loss (6). In animals treated with medetomidine, blood flow is usually directed to vital organs and reduced in muscle, fat, and other non-vital tissues (7).

The pulmonary edema that developed in all RDF animals may also have been conditioned by the parasitic interstitial pneumonia, as lung inflammation predisposes to edema formation (8). Whether the use of medetomidine had also influenced the rapid edema formation is uncertain. In sheep, dexmedetomidine has been shown to induce pulmonary edema in healthy animals (9), but similar findings are not reported in other ruminants.

A prominent pathological finding in all RDF reindeer was a granulomatous pneumonia due to *E. rangiferi* larvae. The adult nematodes live in muscle fasciae and deposit eggs in veins. The eggs are carried to the lungs where they develop to first-stage larvae that penetrate the alveolar wall and enter the air passages, to be carried by mucus up bronchi and trachea, swallowed, and leaving their host through the feces. *E. rangiferi* is prevalent in reindeer in Norway (10), and scattered eggs and larvae are common incidental findings in histological sections from lung tissues of reindeer. Light and moderate infections are not visible macroscopically at necropsy. However, heavy infections, as were evident in the RDF animals, often result in firm “rubbery lungs” that fail to collapse.

Animal R8 (RDR group) had a multifocal purulent bacterial pneumonia when examined *post mortem*. Despite this pneumonia, the chemical immobilization did not cause particular respiratory signs in the second round, 6 days after the first immobilization. The gross and histological findings in the lungs indicated a subacute infection, which might have been a result of RD and aspiration of ruminal content (a common post-anesthetic complication) during the first round of anesthesia. This highlights that any lung condition alone not necessarily causes severe RD during chemical immobilization.

Normal arterial blood oxygen saturation (at sea level) is >95%. Hypoxemia can be defined as mild (90%−95%), marked (75%−90%), and severe (<75%) (2, 11). For the three RDF animals, oxygen saturation in blood (mean SpO_2_ 70%) indicated severe hypoxemia. Also, the RDR animals showed marked or severe hypoxemia during both rounds of immobilization (mean SpO_2_ 77 and 74%, respectively), as compared to the RD– animals (mean SpO_2_ 82 and 86%, respectively), displaying marked but not severe hypoxemia. The use of oxygen supplementation, which is strongly recommended and has been established as a standard procedure (2), would undoubtedly have reduced the hypoxemia for several of these animals. However, due to the per-acute development of RD and pulmonary edema, we conclude that oxygen supplementation would not have been sufficient to save the RDF animals.

Since these animals were immobilized at a resting state, they were not suffering from an increased oxygen demand associated with increased metabolic rate and body temperature, which often is the result of stress and chasing animals prior to darting (2). Further, none of these animals were pregnant or in rut, which represents additional physiological conditions in reindeer that may increase sensitivity to alpha-2 adrenoceptor agonists and contribute to side effects (2, 12). In addition to the ambient temperature, which we have identified as the main variable when comparing the two rounds of immobilization, 11 of the 18 animals that were chemically immobilized a second round showed clinical signs of infectious keratoconjunctivitis (IKC) due to the inoculation with cervid herpesvirus 2 (4). However, since no signs of RD were observed during the second round of immobilization, IKC seemed to have had little or no impact on their general health condition.

It may be controversial to claim that high ambient temperature was an underlying cause of mortality during the immobilization, since there was no registered increase in body temperature. However, the repeated immobilization of the same animals under cooler conditions, using the same drugs and doses for each individual, and without experiencing the same physiological challenges, leads to this suggestion. This further indicates that it is not only the ambient temperature at the actual time of the chemical immobilization that is important, but rather the hours and maybe days prior to the immobilization. Still partly in winter coat and experiencing a sudden high ambient temperature for the season, these animals were suffering from circulatory changes, with blood shunted to the peripheral capillary beds to contribute to loss of excess heat. This already challenged physiological equilibrium may have been further challenged by the adverse cardiovascular effects of medetomidine, causing bradycardia and decrease in cardiac output (13, 14).

It is likely that moderate to severe granulomatous interstitial pneumonia due to *E. rangiferi* larvae and, for 1 animal, airway obstruction from throat bot larvae, may have contributed to the morbidity and mortality during chemical immobilization. During the second round of immobilization, the RDR group still had higher heart rate and lower SpO_2_ than those of the RD– group (Table 1), indicating that RDR animals had functional physiological characteristics that made them more vulnerable to the drugs and doses used, independent of the ambient temperature.

In conclusion, we believe that the main contributing factors to the RD and hypoxemia were high ambient temperature, circulatory collapse and lung edema, and parasite-induced interstitial pneumonia. Although compensated for by the animal under rest, we believe that these conditions were further challenged by the cardiovascular effects of medetomidine. For our study, the availability of live semidomesticated reindeer for purchase was decisive when conducting the immobilization in early June. However, we conclude that chemical immobilization of reindeer with medetomidine–ketamine should be avoided in May–June due to parasites developing in their airways and due to the potential risk when animals partly in winter coats encounter rising ambient temperatures. Supplemental oxygen should be provided during immobilization to compensate for hypoxemia in reindeer immobilized with medetomidine–ketamine.

## Data Availability Statement

The raw data supporting the conclusions of this article will be made available by the authors, without undue reservation.

## Ethics Statement

The animal study was reviewed and approved by Norwegian Animal Research Authority, Forsøksdyrutvalget (FDU), P.O. Box 383, 2381 Brumunddal.

## Author Contributions

MT designed the study. MT, JS, NM, and TM conducted the immobilization. TJ conducted the necropsy and laboratory investigations. MT and TJ wrote the first draft of the manuscript. All authors contributed to the evaluation of the results and to the finalizing of the manuscript and accepted the submitted version.

## Conflict of Interest

The authors declare that the research was conducted in the absence of any commercial or financial relationships that could be construed as a potential conflict of interest.

## Abbreviations

RD: respiratory depression
RDF: respiratory depression with fatal outcome
RDR: respiratory depression, recovered
RD–: no sign of respiratory depression
SpO_2_: oxygen saturation in blood
TBD: time body down
THD: time head down
IKC: infectious keratoconjunctivitis.
